# Exceptionally gigantic aurora in the polar cap on a day when the solar wind almost disappeared

**DOI:** 10.1126/sciadv.adn5276

**Published:** 2024-06-21

**Authors:** Keisuke Hosokawa, Ryuho Kataoka, Takuo T. Tsuda, Yasunobu Ogawa, Satoshi Taguchi, Yongliang Zhang, Larry J. Paxton

**Affiliations:** ^1^ Graduate School of Communication Engineering and Informatics, University of Electro-Communications, Tokyo 182-8585, Japan.; ^2^ Center for Space Science and Radio Engineering, University of Electro-Communications, Tokyo 182-8585, Japan.; ^3^ National Institute of Polar Research, Tokyo 190-0014, Japan.; ^4^ Graduate School of Science, Kyoto University, Kyoto 606-8502, Japan.; ^5^ The Johns Hopkins University Applied Physics Laboratory, Laurel, MD 20723, USA.

## Abstract

Revealing the origins of aurorae in Earth’s polar cap has long been a challenge since direct precipitation of energetic electrons from the magnetosphere is not always expected in this region of open magnetic field lines. Here, we introduce an exceptionally gigantic aurora filling the entire polar cap region on a day when the solar wind had almost disappeared. By combining ground-based and satellite observations, we proved that this unique aurora was produced by suprathermal electrons streaming directly from the Sun, which is known as “polar rain.” High-sensitivity imaging from the ground has visualized complex spatial structures of the polar rain aurora possibly manifesting the internal pattern of the solar wind or even the organizations in the chromosphere of the Sun.

## Teaser

Bright aurora filling the polar cap of Earth was caused by hard rain of energetic electrons directly coming from the Sun.

## INTRODUCTION

An aurora is a fascinating luminous phenomenon in Earth’s upper atmosphere at altitudes of 100 to 300 km (*1*). Discrete-type aurorae are characterized by their highly variable nature and highly structured appearance (*2*), while diffuse-type aurorae show dynamically changing features such as pulsation (*3*). Those auroral emissions are released by atoms and molecules excited by energetic electrons precipitating from the magnetosphere along the closed magnetic field lines of the planet. Those incident electrons are stored in the magnetotail, energized by solar wind forcing, and then precipitated into the atmosphere in the manner of a burst known as an auroral substorm (*1*, *4*). Thus, the driver of Earth’s aurorae is not always a direct supply of electrons from the Sun.

**Fig. 1.**
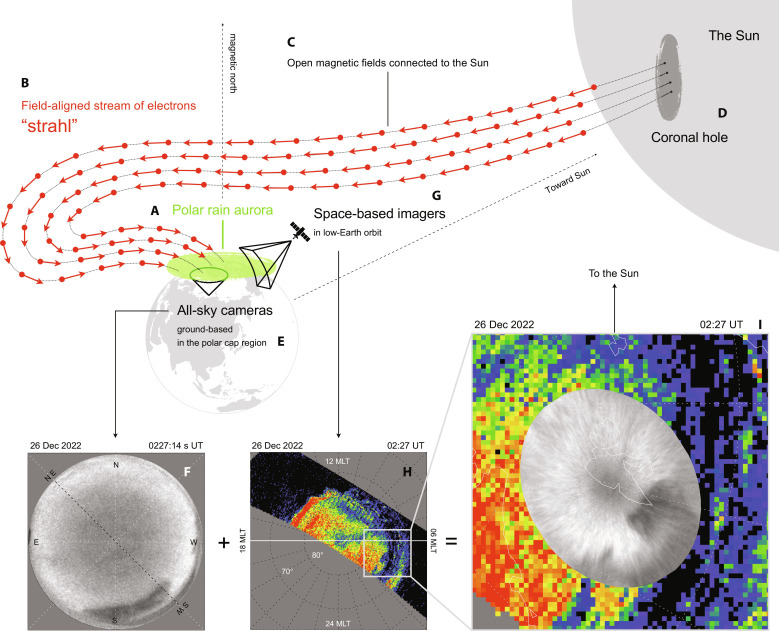
Schematic of the relationship between the strahl electrons from the Sun and the polar rain aurora in the polar cap. (**A**) Polar rain aurora in the Northern Hemisphere covering the entire polar cap region. (**B**) Magnetic field–aligned stream of suprathermal electrons from the Sun called strahl electrons. (**C**) Open geomagnetic field lines from the northern polar cap connected to the interplanetary magnetic field (IMF) originating from the Sun. The strahl electrons travel along these connected field lines from the Sun to Earth’s polar cap. (**D**) Coronal hole on the surface of the Sun, which is one of the sources of the open field lines and the strahl electrons although open magnetic fluxes also originate from coronal streamers. (**E**) All-sky cameras (ASCs) located at the polar cap latitudes in Longyearbyen, Svalbard Island, Norway. (**F**) Green line all-sky image at 557.7 nm taken by the electron multiplying charge-coupled device (EMCCD) camera in Longyearbyen at 02:27 UT on 26 December 2022. (**G**) Space-based imagers onboard the low-altitude polar-orbiting Defense Meteorological Satellite Program (DMSP) satellites. (**H**) Large-scale image of aurora in the ultraviolet (UV) band taken by the Special Sensor Ultraviolet Scanning Imager (SSUSI) instrument onboards the DMSP satellite at around 02:27 UT on 26 December 2022. The optical data have been mapped onto the magnetic latitude (MLAT)/magnetic local time (MLT) polar coordinate system. (**I**) Zoomed-in view of combined images of ground-based and space-based optical data at 02:27 UT on 26 December 2022. The data from the ground-based EMCCD camera were mapped onto the MLAT/MLT coordinate system, assuming an emission height of 110 km.

There is one specific type of aurora that is caused by electrons traveling directly from the Sun’s corona. Such a rare aurora, imaged only a small number of times by satellites, manifests the so-called “polar rain” electron precipitation in the polar cap (*5*, *6*). The source of this polar rain and the corresponding “polar rain aurora” (Fig. 1A) is field-aligned “strahl” electrons (Fig. 1B), which represent a stream of suprathermal electrons traveling from the Sun along open magnetic field lines (Fig. 1C) that connect Earth’s polar cap with the coronal hole on the surface of the Sun (Fig. 1D) (*7*). Intense polar rain precipitation tends to occur when the solar wind density is low, which means that Coulomb scattering interference with the field-aligned travel of strahl electrons from the Sun is minimized (*8*).

Polar rain has been studied extensively using low-altitude satellite particle measurements (*9*–*13*). In contrast, few studies have investigated polar rain aurorae because of the limited number of detectable cases (*5*, *6*). In addition, only some ground-based optical observation stations, sufficiently sensitive to the weak emissions caused by polar rain precipitation, have been operative at high latitudes, especially in the polar cap. For these reasons, no single case of polar rain aurora has been detected from the ground; thus, the polar rain aurora has yet to be recognized as one of the major classes of aurorae in the polar cap (*14*). Moreover, the insufficient spatiotemporal resolution of satellite imagery has also made it difficult to investigate the spatial structure and dynamical characteristics of the polar rain aurora.

## RESULTS

### Gigantic aurora in the polar cap

On 25 to 26 December 2022, a peculiar auroral form, which was possibly a manifestation of the polar rain aurora, was captured by ground-based all-sky cameras (ASCs) in the polar cap (Fig. 1E). Figure 1F displays a representative all-sky image of 557.7-nm emissions from oxygen atoms taken by an all-sky electron multiplying charge-coupled device (EMCCD) camera in Longyearbyen, Norway (*15*). This auroral form was astonishingly uniform (i.e., smooth), and its spatial extent was far beyond the field of view of the ground-based camera. This incredibly smooth and gigantic form is distinctively different from that of a typical polar cap aurora (*14*); thus, it cannot be categorized as any previously identified class of aurorae visible at polar cap latitudes. One plausible candidate phenomenon is the polar rain aurora, which has never previously been observed from the ground.

To confirm this possibility, we checked that auroral images from the Special Sensor Ultraviolet Scanning Imager (SSUSI) (*16*) onboard the polar-orbiting Defense Meteorological Satellite Program (DMSP) satellites (Fig. 1G). Figure 1H displays one such satellite image in the Lyman-Birge-Hopfield short band in the ultraviolet (UV) wavelength, obtained simultaneously with the ground-based imaging (Fig. 1F). Almost the entire part of the polar cap, above approximately 75 magnetic latitudes (MLATs), was filled with less-structured smooth emissions, which strongly resembled the limited cases of polar rain aurorae observed in the past (*5*, *6*). The spatial structure of this peculiar aurora seen from the ground shows overall good agreement with that of the polar rain aurora seen from space (Fig. 1I). Movie S1 displays all the combined ground-based and space-based optical observations acquired on 25 to 26 December 2022. Although the agreement between the two datasets is generally good, the much-improved spatial resolution of the ground-based data enables us to reveal the fine-scale structure and dynamical characteristics of the polar rain aurora.

**Fig. 2.**
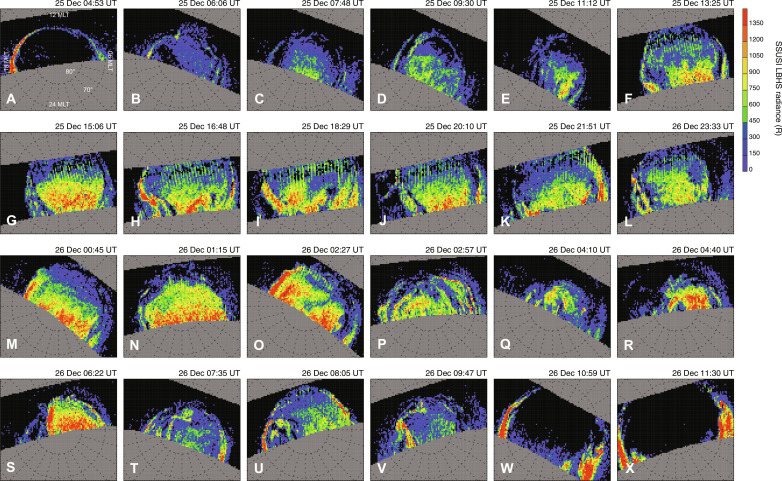
Sequence of large-scale auroral images from the SSUSI/DMSP in the Northern Hemisphere on 25 to 26 December 2022. (**A**) Image at 04:53 UT on 25 December 2022, when the polar cap was dim and surrounded by the distinct ring of the auroral oval. (**B** to **F**) Images taken from 06:06 to 13:25 UT on 25 December 25 2022, when the polar cap was slowly filled with diffuse auroral emissions. (**G** to **S**) Images from 15:06 UT on 25 December to 06:22 UT on 26 December 2022, when the polar cap was almost entirely filled with intense diffuse auroral emissions. (**T** to **V**) Images from 07:35 to 09:47 UT on 26 December 2022, when the diffuse emissions in the polar cap gradually faded. (**W** and **X**) Images at 10:59 and 11:30 UT on 26 December 2022, when the polar cap had returned to its original configuration, i.e., a dim polar cap surrounded by the auroral oval. All images were mapped onto the MLAT/MLT polar coordinate system. The center of each panel corresponds to the geomagnetic pole in the Northern Hemisphere, and the dayside (12 MLT, i.e., the direction of the Sun) is oriented to the top. LBHS, Lyman-Birge-Hopfield Short.

Figure 2 shows a sequence of satellite images from the DMSP/SSUSI from 04:53 UT on 25 December to 11:30 UT on 26 December 2022. Figure 2A represents the normal distribution of the aurora, where the entirely empty polar cap region is surrounded by the ring-shaped bright auroral oval at lower latitudes. At ~06 UT on 25 December (Fig. 2B), the polar cap started to become filled with a faint diffuse aurora. Subsequently, almost the entire polar cap region soon became covered by intense but less-structured emissions (e.g., Fig. 2, G and N). This large-scale filling of the polar cap by diffuse aurorae continued for ~28 hours from 06 UT on 25 December to ~10 UT on 26 December (Fig. 2, B to V). At ~07:30 UT on 26 December (Fig. 2T), the intense emission inside the polar cap started to fade, and within a few hours, the structure of the aurora had returned to the normal distribution characterized by the empty polar cap surrounded by the ring-shaped auroral oval (Fig. 2, W and X).

### Polar rain aurora when the solar wind almost disappeared

**Fig. 3.**
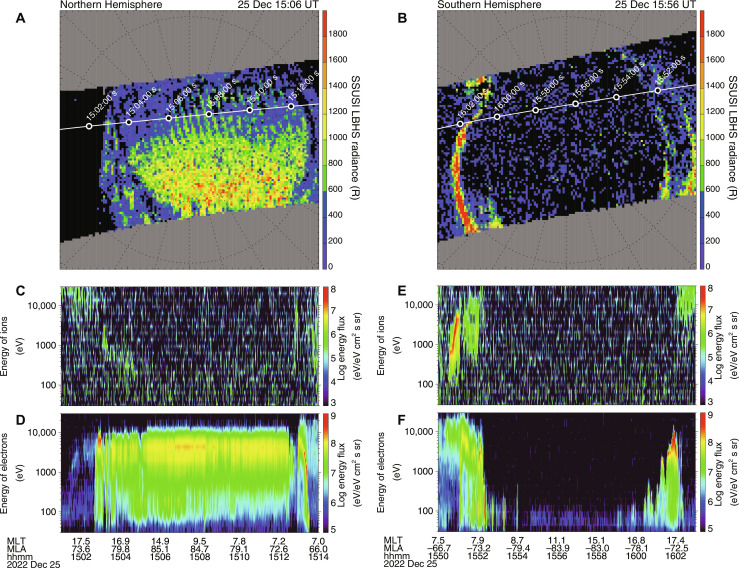
Optical and particle observations by the DMSP satellites in both hemispheres. (**A**) UV image from the SSUSI onboard the DMSP F17 satellite in the Northern Hemisphere taken at ~15:06 UT on 25 December 2022. Almost the entire polar cap was filled with diffuse and less-structured emissions of the polar rain aurora. The trajectory of the DMSP F17 satellite is overplotted. (**B**) UV image from the SSUSI onboard the DMSP F17 satellite in the Southern Hemisphere taken at ~15:56 UT on 25 December 2022. The polar cap was almost empty, surrounded by the bright ring of the auroral oval at lower latitudes. (**C** and **D**) Energy-time spectrogram of ions (C) and electrons (D) taken by the particle instruments onboard the DMSP F17 satellite during the interval plotted in (A). In (D), intense fluxes of precipitating electrons, whose energy is in the range from 100 eV up to 10 keV (the characteristic energy is ~5 keV), were observed when the satellite passed through the central polar cap, i.e., the region of the polar rain aurora. This electron precipitation produced the optical signature of the polar rain aurora displayed in (A). (**E** and **F**) Energy-time spectrogram of ions (E) and electrons (F) taken by the particle instruments onboards the DMSP F17 satellite during the interval plotted in (B). Any signatures of electron precipitation are not seen in the central polar cap (i.e., in the middle of the energy-time spectrogram), consistent with the absence of the polar rain aurora in the Southern Hemisphere (B).

**Fig. 4.**
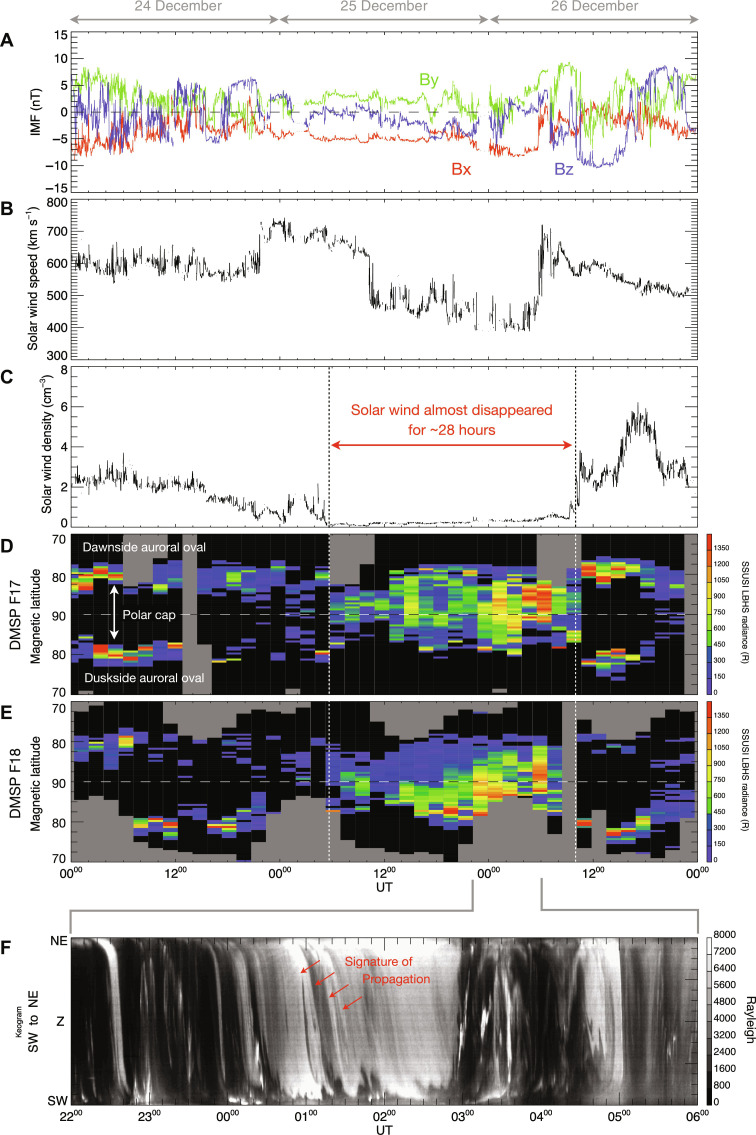
Relationship between the upstream solar wind conditions and the polar rain aurora seen from space and the ground. (**A**) The three components of the IMF (Bx, red; By, green; Bz, blue) from 00:00 UT on 24 December to 24:00 UT on 26 December 2022. (**B**) The solar wind speed. (**C**) The solar wind proton density. (**D**) Temporal variation in the UV aurora taken by the SSUSI onboard the DMSP F17 satellite presented in the format of a keogram. The optical data sampled along the dawn-dusk cross section are plotted as a function of time. (**E**) Same as (D) but using data from the SSUSI onboard the DMSP F18 satellite. (**F**) Temporal variation in the 557.7-nm EMCCD ASC data from Longyearbyen sampled along a southwest (SW)–northeast (NE) cross section from 22:00 UT on 25 December to 06:00 UT on 26 December 2022.

Complete filling of the polar cap was not recognized in the DMSP/SSUSI images from the Southern Hemisphere (Fig. 3B), whereas an image obtained almost simultaneously from the Northern Hemisphere shows a clear signature of the polar rain aurora (Fig. 3A). Polar rain precipitation generally occurs in only one hemisphere depending on the orientation of the interplanetary magnetic field (IMF) (*7*, *10*). If the IMF is directed toward (away from) Earth, then the polar rain aurora is observed only in the Northern (Southern) Hemisphere. This is because the field lines of the IMF directed toward (away from) Earth can be connected to the open field line from the polar cap in the Northern (Southern) Hemisphere through magnetic reconnection in the high-latitude part of the magnetotail (*7*). Figure 4A illustrates the three components of the IMF on 24 to 26 December 2022. The IMF Bx (red line in Fig. 4A) was negative, i.e., directed toward Earth, which is a condition favorable for guiding strahl electrons from the Sun toward the northern polar cap along the reconnected field lines. The observed interhemispheric asymmetry and the expected orientation of the IMF strongly suggest that the large-scale auroral feature detected inside the polar cap was a manifestation of the polar rain aurora.

This remarkable interhemispheric asymmetry was also seen in the particle observations from the DMSP satellites (Fig. 3, C to F), where intense polar rain precipitation was seen only in the electron data from the Northern Hemisphere (Fig. 3D). The characteristic energy of this precipitation was ~5 keV, i.e., relatively high compared with that of typical polar rain cases (*12*). This more-energetic polar rain precipitation tends to occur during intervals of low solar wind density (*8*). The solar wind parameters for 24 to 26 December 2022 are shown in Fig. 4 (B and C). On 25 to 26 December, there was a discrete ~28-hour interval during which “the solar wind almost disappeared,” when the solar wind density (Fig. 4C) was extremely low, sometimes below 0.5 cm^−3^. Data from the SSUSIs onboard the DMSP F17 and F18 satellites, presented in Fig. 4 (D and E), respectively, are plotted in the format of a keogram, which is a time series of optical data sampled along the dawn-dusk cross section indicated by the horizontal dashed line in Fig. 1H. Before and after the interval of low solar wind density, the polar cap was dim and surrounded by the auroral oval on the dawn/dusk sides. In contrast, when the solar wind almost disappeared, the polar cap became filled with intense auroral emissions. This excellent correlation between the timing of the solar wind disappearance and that of the filling of the polar cap proves that the smooth and gigantic aurora captured from the ground manifested the exceptionally intense polar rain aurora that occurred when the solar wind almost disappeared.

### First detection of polar rain aurora from the ground

Figure 4F illustrates the temporal variation of the optical data from Longyearbyen from 22 UT on 25 December to 06 UT on 26 December, when the polar rain aurora imaged by the DMSP/SSUSI was most prominent. Here, the 557.7-nm oxygen emissions are plotted in the format of a keogram along a southwest-northeast cross section (dashed line in Fig. 1F), which is broadly aligned with the Sun-Earth line in the polar cap. During an approximate 3-hour period in the middle of the interval, bright emissions with absolute optical intensity as high as 8 kR were identified. Typical auroral arcs in the polar cap are characterized by reddish emissions at 630.0 nm caused by less-energetic electrons (<1 keV) (*14*). In the current case, however, the greenish emission at 557.7 nm was dominant in the color images acquired using a different digital camera in the same location (fig. S1), implying that more energetic electrons formed the primary component of the polar rain precipitation. When the solar wind disappeared, an intense flux of electrons with an energy of >1 keV was observed by the DMSP (Fig. 3D), which made the polar rain aurora visible even from the ground as bright greenish emissions.

The cross-sectional optical data plotted in Fig. 4F contain a few diagonal traces elongated from the northeast toward the southwest, representing propagation of the polar rain aurora in the anti-sunward direction. Movie S2 shows more clearly such anti-sunward motion of the polar rain aurora on which the motion vectors estimated through two-dimensional (2D) cross-correlation of consecutive images (*17*, *18*) are superimposed. The estimated speed varied in time, ranging from 200 to 1000 m s^−1^ (mean, 399 ± 175 m s^−1^) (fig. S2D). The direction of propagation was generally anti-sunward (fig. S2E). A previous study using satellite images (*5*) demonstrated that the polar rain aurora moves anti-sunward with speed of 150 m s^−1^. The estimated speed of the polar rain aurora as viewed from the ground was two to three times faster than that of the previous case and approximately as fast as the average speed of plasma convection in the polar cap (*17*, *18*). During this interval, the plasma convection speed estimated from the cross polar cap potential from the Super Dual Auroral Radar Network (*19*) was ~400 m s^−1^. This value is consistent with the average speed of the polar rain aurora shown in fig. S2D, implying that the anti-sunward propagation of the polar rain aurora represents the motion of magnetic flux tubes connecting the polar cap with the surface of the Sun. Thus, ground-based images of the polar rain aurora could be used to visualize how the streaming solar wind might drag such magnetic flux tubes.

## DISCUSSION

The polar rain aurora, which was distributed uniformly over the entire polar cap (e.g., Fig. 2N), had horizontal scale of up to 4000 km. This spatial extent could represent the scale of a bundle of open magnetic flux tubes having similar characteristics. The origin of the open magnetic field from the coronal hole is considered the so-called “magnetic funnel base” at the boundaries of supergranules in the photosphere (*20*–*22*). The diameter of a bundle of open flux tubes originating from a magnetic funnel base with a diameter of 150 km (*23*–*26*) can be estimated as ~7500 km when projected onto Earth’s polar cap. This value is much larger than the scale of the current polar rain aurora of ~4000 km, explaining its exceptionally smooth distribution over almost the entire polar cap.

**Fig. 5.**
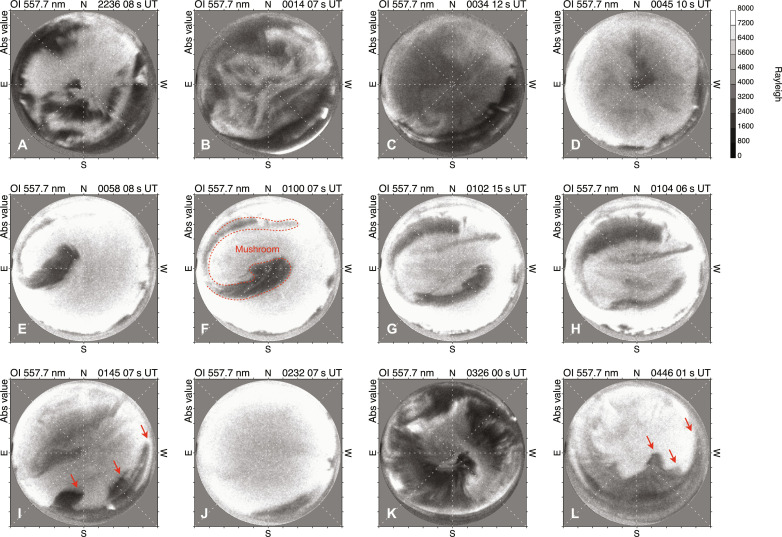
Collection of ground-based views of the polar rain aurora in the 557.7-nm auroral images from the EMCCD ASC in Longyearbyen. (**A** to **D**) Representative images from 22:36 UT on 25 December to 00:45 UT on 26 December 2022. In (A) and (B), some patchy and diffuse substructures of the polar rain aurora can be identified. In (C) and (D), the polar rain aurora was spatially uniform. (**E** to **H**) Four consecutive images from 00:58 to 01:04 UT on 26 December 2022. The polar rain aurora was again spatially uniform, but its boundary showed large-scale undulation forming a mushroom-like structure. This feature drifted from the northeast toward the southwest (top left to bottom right in the figures), corresponding to the anti-sunward motion in the polar cap. (**I** to **L**) Representative images from 01:45 to 04:46 UT on 26 December 2022. In (J), the polar rain aurora was again extremely smooth in shape. In (I) and (L), undulating wavy features can be seen, as indicated by the red arrows.

A previous study based on particle observations reported the existence of substructures with scale of ~1700 km within polar rain precipitation (*27*). The detailed ground-based view of the polar rain aurora revealed the existence of further smaller-scale features, which are presented in Fig. 5. The structure of the polar rain aurora distributed over the entire field of view was mostly smooth (e.g., Fig. 5, C, D, and J). However, complex internal structures were often seen, such as those composed of relatively small patches of diffuse aurora (Fig. 5, A, B, and K). Furthermore, consecutive images at 2-min intervals (Fig. 5, E to H) demonstrate a large mushroom-like feature moving anti-sunward. Similar but smaller undulations of the boundary were also seen at later times (indicated by red arrows in Fig. 5, I and L). These small-scale structures and characteristic boundary shapes might represent the cross-sectional pattern of open flux tubes in the solar wind (*22*) or the shape of the origin of those flux tubes, i.e., magnetic funnel bases in the photosphere of the Sun. Moreover, all or at least part of the structures could have been produced by Kelvin-Helmholtz or Rayleigh-Taylor plasma instability at the high-latitude magnetopause (*6*, *27*) or in the magnetosphere-ionosphere coupling system (*28*). At this stage, however, it remains difficult to pinpoint how and where the internal structures of the polar rain aurora were formed on the way from the solar surface to Earth’s polar cap.

We captured an extremely bright and gigantic polar rain aurora during a period of disappearance of the solar wind, which was visible even from the ground. The improved spatiotemporal resolution of ground-based instruments allowed us to visualize the dynamic motion and complex internal structures embedded within the large-scale feature. The formation of such structures might be attributable to plasma instability at the interface between the magnetosphere and the solar wind (*27*) or directly related to the spatial structure of the coronal hole solar wind (*22*). If the latter hypothesis is correct, then the polar rain aurora simply projects patterns in the solar wind or on the solar surface as a screen; thus, ground-based ASCs at the polar cap latitudes could be used for 2D remote sensing of structures (e.g., supergranules, magnetic funnel bases, and magnetic network lanes) in the photosphere of the Sun, which is the origin of the internal structures of the solar wind. In either case, the fine-scale forms of the polar rain aurora found in this study pose new puzzles and challenges in further investigation of the Sun-Earth connection. The true nature of the magnetic flux tubes, connecting the vast region extending from the solar surface to Earth’s polar cap, should be diagnosed through extremely high-resolution future magnetohydrodynamic modeling developed to reproduce all the combined processes.

## MATERIALS AND METHODS

### Ground-based optical observations in the polar cap

This study used ground-based optical observations obtained using two ASCs operated at the Kjell Henriksen Observatory in Longyearbyen, Svalbard, Norway (78.148°N, 16.043°E). The EMCCD ASC (*17*), used for the core analysis, comprised a Hamamatsu Photonics C9100-13 EMCCD camera with ASI-2 optics (Nikon Ltd.) (*29*). The C9100-13 EMCCD camera has spatial resolution of 512 pixels by 512 pixels. The ASI-2 optics unit has two narrow-band interference filters at 557.7 and 630.0 nm for airglow/aurora observations. This study used the 557.7-nm filter to observe green line oxygen emissions at altitudes of approximately 110 km. The full width at half maximum of the optical filters was ~3 nm, and the exposure time for the 557.7 nm observations was 2 s. During the interval of the present study (25 to 26 December 2022), the EMCCD ASC was operated continuously, even during daytime, because both the Sun and the Moon were below the elevation angle threshold, which allowed us to observe the signature of the polar rain aurora in all the local time sectors.

The ASI-2 optics also observed the background continuum emission at 572.5 nm, where no prominent airglow/auroral emission line exists, which was used for deriving the absolute optical intensity in units of Rayleigh. We observed those three emission lines by rotating the filter turret attached to the ASI-2 optics. The average temporal resolution of the 557.7 nm emission was 20 s during the interval of the present study (i.e., the ASC captured three green line images per minute). The EMCCD ASC was calibrated using the integrating sphere of the National Institute of Polar Research of Japan (*30*). Combining the calibration data with the background continuum emission acquired every 20 min, the absolute optical intensity of the 557.7-nm emission was derived (*31*) and used for quantitative analysis of auroral emission intensity.

The other ASC system comprised the so-called “Raspberry Pi” ASC (Raspberry Pi high-quality camera, Raspberry Pi Ltd.), which is a color digital camera operated by a single-board computer (Raspberry Pi 3B+, Raspberry Pi Ltd.). A fish-eye lens (TC1514HD-IR, Kenko Tokina Co. Ltd.) was mounted onto the camera. The Raspberry Pi ASC was used mainly for inferring the actual color of the polar rain aurora. The resolution of the imaging sensor (IMX477R, Sony) of the camera is 4056 pixels by 3040 pixels. The exposure time of data capture was 21 s during nighttime, and images were obtained at 30-s intervals. The color images were saved as processed jpeg files in three channels (i.e., red-green-blue channels) with 8-bit depth. 

### DMSP SSUSI instrument

To capture the large-scale structure of the polar rain aurora, we exploited the SSUSIs (*16*, *32*, *33*) onboard the DMSP F17 and F18 satellites. Large-scale images of aurora in the Lyman-Birge-Hopfield short channel (140 to 150 nm), obtained from horizon-to-horizon line-scanning observations in the imaging mode, were used for the current study. The DMSP satellites are in Sun-synchronous polar orbit at an altitude of approximately 850 km with orbital period of ~97 min. The orbits of the DMSP F17 and F18 satellites were broadly aligned with the dawn-dusk meridian during the studied interval; therefore, large-scale images of aurora in the polar cap were obtained at approximately 50-min intervals. Because of the substantial difference in temporal resolution between the EMCCD ASC (20 s) and the SSUSI (~50 min), the agreement between the two sets of optical data was not always perfect. We used absolute optical intensity data in units of Rayleigh, derived by removing any possible contamination of dayglow in the dayside part of the field of view of the SSUSI. Because of their use, the data used in this study were mapped onto and plotted in the polar coordinate system based on the altitude-adjusted corrected geomagnetic coordinates MLAT/magnetic local time (MLT). The spatial resolution of the mapped SSUSI data is ~0.25° in the latitudinal direction.

### Estimation of propagation velocity of polar rain aurora

The propagation velocity of the polar rain aurora was estimated using an object-tracking algorithm based on the cross-correlation between two consecutive images separated by an interval of 20 s. This method was initially developed to track the motion of upper-atmospheric phenomena in the 630.0-nm airglow emission at polar cap latitudes, called polar cap patches (*17*, *18*). When tracking the movement of the polar rain aurora, consecutive pairs of original 557.7-nm all-sky images (512 pixels by 512 pixels) were processed according to the following steps, which represent slight modification of the original procedure. (i) Twenty-five (5 × 5) reference points are defined near the zenith of the field of view (25 red points near the center of fig. S2A). (ii) The image is divided into 80-pixel by 80-pixel template windows by setting the reference point as a center. (iii) The template window is shifted in the succeeding image, and cross-correlation coefficients are calculated between the template and all shifted windows. (iv) The drift velocity is calculated using the relative displacement vector from the template window to the shifted one where the maximum cross-correlation coefficient is obtained. These displacement vectors are shown in fig. S2A by red arrows. The actual drift velocities were estimated by mapping the displacement vectors in the geographic coordinate system by assuming the emission height of 110 km. Those velocity vectors, further mapped onto the MLAT/MLT polar coordinate system, are overplotted onto the mapped all-sky image in fig. S2B. Note that vectors with low cross-correlation coefficients (<0.6) are not presented (the points of the open circle). The time series of the speed and angle of the drift velocity, plotted in fig. S2 (D and E), respectively, were computed by averaging the 25 estimated displacement vectors. The average drift speed during the 2-hour interval from 00:00 to 02:00 UT on 26 December 2022 was estimated at 399 ± 175 m s^−1^.

### Estimation of the plasma convection speed in the polar cap

During the studied interval, the cross polar cap potential derived from the Super Dual Auroral Radar Network (*19*) was ~50 kV. Because the distance between the maximum and the minimum of the electric potential distribution was ~2000 km, the average electric field magnitude should be ~25 mV m^−1^ in the polar cap. The magnitude of the *E* × *B* drift velocity *v* can be calculated as *v* = *E*/*B*, i.e., ~400 m s^−1^, if we assume the intensity of the magnetic field in the polar cap to be 6 × 10^5^ nT. This value is close to the average speed of the polar rain aurora shown in fig. S2D.

### Connection of the polar rain aurora with structures on the Sun

The scale size of the magnetic funnel bases in the photosphere of the Sun, seen as a bright point in the G band (4305 A) image of the Sun (*22*), *D*_0_, is ~150 km in the horizontal direction (*23*, *24*), and the intensity of the magnetic field within the bright point *B*_0_ is approximately 0.15 T (*22*, *25*). Considering the conservation of the total magnetic flux within the open magnetic flux tubes from a single magnetic funnel and assuming the magnetic field intensity in Earth’s polar cap *B*_1_ to be ~6 × 10^−5^ T, the diameter of the bundle of the flux tubes corresponding to the magnetic funnel base with diameter *D*_0_ can be estimated at *D*_1_ = *D*_0_ √ (*B*_1_/*B*_0_), i.e., ~7500 km, at the end point on Earth side (i.e., in the polar cap). This value is much larger than the scale size of the polar rain aurora of ~4000 km, which can explain the exceptionally smooth distribution within the polar rain aurora over a wide area. Moreover, this estimation implies the possibility that the small-scale structures embedded within the polar rain aurora represent the cross-sectional pattern within the bundle of the open flux tubes or the shape of the origin of those flux tubes, i.e., magnetic funnel bases in the photosphere.
