# Supplementary material for: Exceptionally gigantic aurora in the polar cap on a day when the solar wind almost disappeared
Source: Sci Adv. 2024 Jun 21;10(25):eadn5276. doi: 10.1126/sciadv.adn5276 (PMC11192072; doi:10.1126/sciadv.adn5276)
Supplement: Supplementary file 1 — Figs. S1 and S2 Legends for movies S1 and S2 [file sciadv.adn5276_sm.pdf]

Science Advances
AAAS

Supplementary Materials for

Exceptionally gigantic aurora in the polar cap on a day when the solar wind almost disappeared

Keisuke Hosokawa *et al.*

Corresponding author: Keisuke Hosokawa, keisuke.hosokawa@uec.ac.jp

Sci. Adv. **10**, eadn5276 (2024)
DOI: 10.1126/sciadv.adn5276

The PDF file includes:

Figs. S1 and S2
Legends for movies S1 and S2

Other Supplementary Material for this manuscript includes the following:

Movies S1 and S2

Fig. S1.

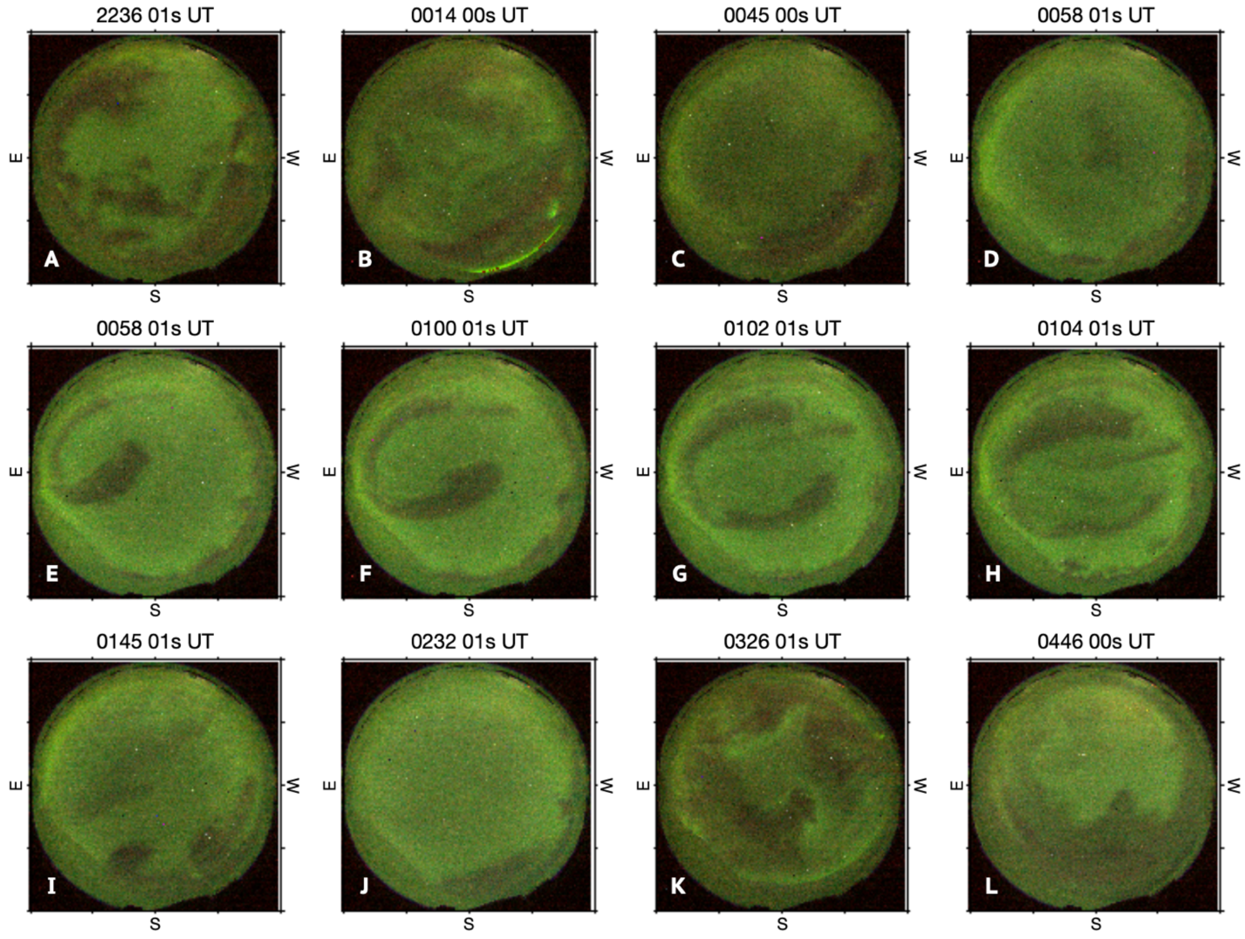

Fig. S1. Collection of ground-based all-sky images from the colour digital camera taken in Longyearbyen. (**A**–**L**) Representative colour images of the polar rain aurora on 25–26 December 2022. The timings of these images are the same as those of the images shown in Fig. 5. The dominant green colour of the auroral emissions indicates that the polar rain aurora during the interval of interest was caused by precipitation of energetic electrons whose energy was at least >1 keV. This is consistent with the intense precipitating flux of ~5 keV electrons seen in Fig. 3.

Fig. S2.

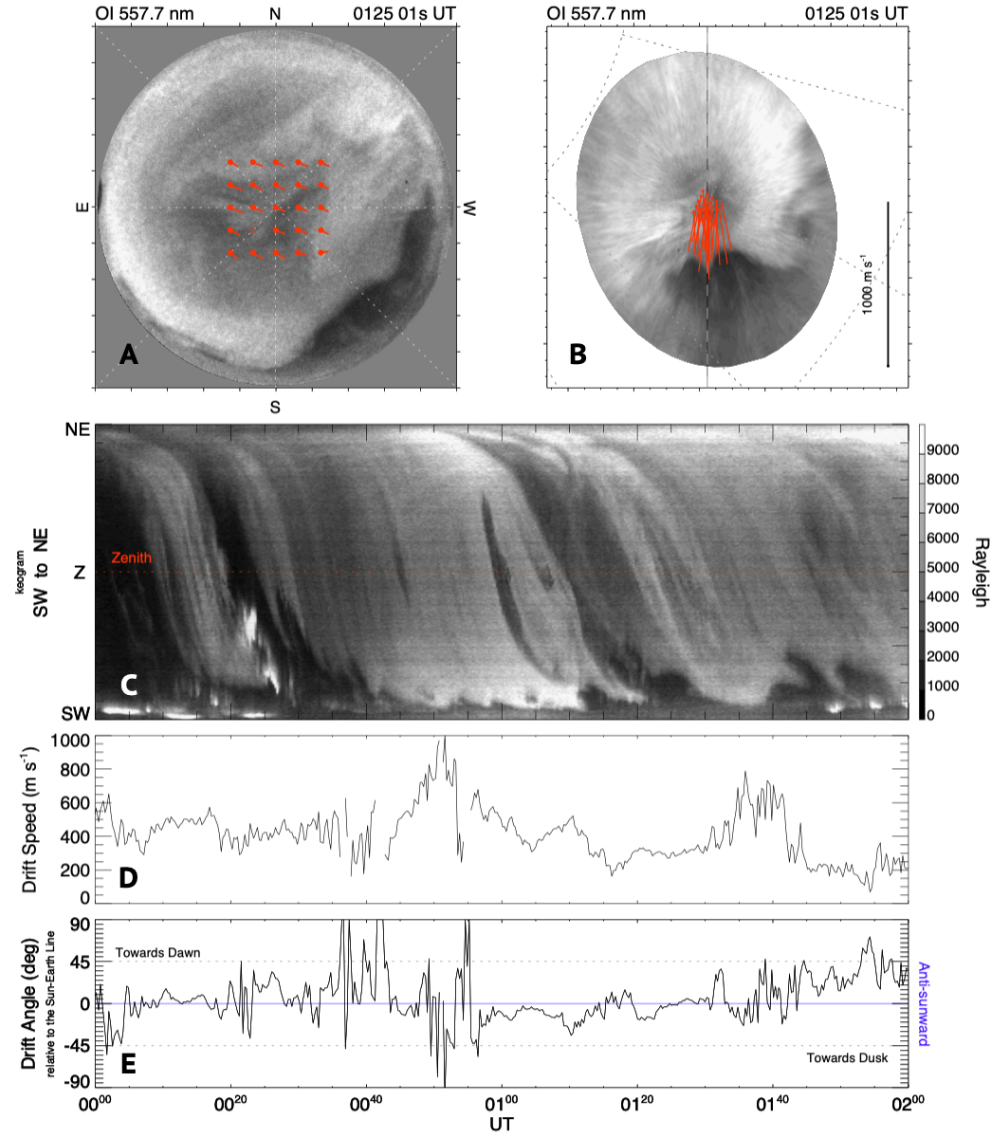

Fig. S2. Tracking the motion of the polar rain aurora. (**A**) An all-sky image at 01:25 UTC on 26 December 2022, with the displacement vectors (motion vectors) computed by the two-dimensional cross-correlation analysis superimposed. (**B**) Same as in (A) but the optical data and the derived displacement vectors have been mapped onto the Magnetic Latitude (MLAT)/Magnetic Local Time (MLT) polar coordinate system by assuming an emission altitude of 110 km. The direction of the Sun is toward the top of the figure, and the vertical dashed line shows the Sun–Earth line in the polar cap. (**C**) SW–NE keogram of the 557.7 nm emission during 00:00–02:00 UTC on 26 December 2022, in which several oblique traces representing the anti-sunward propagation of the polar rain aurora are seen. (**D**) Speed of the drift of the polar rain aurora estimated from the cross-correlation analysis. The average drift speed during this 2-h interval was estimated at 399 ± 175 m s^{-1}. (**E**) Angle of the drift of the polar rain aurora. The direction of the drift is presented as an angle relative to the Sun–Earth line in the polar coordinate system, and it was predominantly anti-sunward throughout the studied interval.

Movie S1.

Overview of the optical observations from space and the ground. (**Left**) 557.7 nm all-sky images obtained by the electron multiplier charge-coupled device (EMCCD) all-sky camera in Longyearbyen from 00:00 UTC on 25 December to 09:40 UTC on 26 December 2022. From approximately 13 UTC on 25 December 2022 to 05 UTC on 26 December 2022, the signature of the polar rain aurora was seen almost continuously as diffuse/less structured emissions. (**Centre**) Same all-sky image as in the left panel but mapped onto the Magnetic Latitude (MLAT)/Magnetic Local Time (MLT) polar coordinate system assuming an altitude of auroral emission at 110 km. The optical data from the Special Sensor Ultraviolet Scanning Imager (SSUSI) instruments onboard the Defense Meteorological Satellite Program (DMSP) F17 and F18 satellites are also plotted in the background. Overall agreement between the ground-based and space-based optical data is evident. (**Right**) Same as in the central panel but plotted for the entire polar region in the Northern Hemisphere. The large-scale structure of the polar rain aurora was seen to fill most parts of the polar cap throughout most of this extended interval.

Movie S2.

Estimation of the motion vector of the polar rain aurora. (**Left**) All-sky images from 00:00 to 02:00 UTC on 26 December 2022, where the displacement vectors (motion vectors) obtained from the two-dimensional cross-correlation analysis are overplotted by the red arrows. When the small-scale structure of the polar rain aurora is seen near the zenith of the all-sky image, the estimated displacement vectors well track the actual motion of the emission. (**Right**) Same data as in the left panel but the optical data and derived motion vectors have been mapped onto the Magnetic Latitude (MLAT)/Magnetic Local Time (MLT) polar coordinate system by assuming an emission altitude of 110 km. The direction of the Sun is toward the top of the figure, and the vertical white line shows the Sun–Earth line in the polar coordinate system. The estimated motion vectors were directed predominantly downward in the figure, indicating anti-sunward motion of the polar rain aurora.
